# Female dietary patterns and outcomes of in vitro fertilization (IVF): a systematic literature review

**DOI:** 10.1186/s12937-021-00757-7

**Published:** 2022-01-18

**Authors:** Elizabeth A. Sanderman, Sydney K. Willis, Lauren A. Wise

**Affiliations:** 1 Boston College, Boston, USA; 2 Boston University School of Public Health, Boston, USA

**Keywords:** Dietary patterns, Diet, IVF, In vitro fertilization, Assisted reproductive technology, ART, Infertility, Fertility, Female, Maternal

## Abstract

**Background:**

Infertility affects up to 15% of couples. In vitro fertilization (IVF) treatment has modest success rates and some factors associated with infertility and poor treatment outcomes are not modifiable. Several studies have assessed the association between female dietary patterns, a modifiable factor, and IVF outcomes with conflicting results. We performed a systematic literature review to identify female dietary patterns associated with IVF outcomes, evaluate the body of evidence for potential sources of heterogeneity and methodological challenges, and offer suggestions to minimize heterogeneity and bias in future studies.

**Methods:**

We performed systematic literature searches in EMBASE, PubMed, CINAHL, and Cochrane Central Register of Controlled Trials for studies with a publication date up to March 2020. We excluded studies limited to women who were overweight or diagnosed with PCOS. We included studies that evaluated the outcome of pregnancy or live birth. We conducted an initial bias assessment using the SIGN 50 Methodology Checklist 3.

**Results:**

We reviewed 3280 titles and/or titles and abstracts. Seven prospective cohort studies investigating nine dietary patterns fit the inclusion criteria. Higher adherence to the Mediterranean diet, a ‘profertility’ diet, or a Dutch ‘preconception’ diet was associated with pregnancy or live birth after IVF treatment in at least one study. However, causation cannot be assumed. Studies were potentially hindered by methodological challenges (misclassification of the exposure, left truncation, and lack of comprehensive control for confounding) with an associated risk of bias. Studies of the Mediterranean diet were highly heterogenous in findings, study population, and methods. Remaining dietary patterns have only been examined in single and relatively small studies.

**Conclusions:**

Future studies with rigorous and more uniform methodologies are needed to assess the association between female dietary patterns and IVF outcomes. At the clinical level, findings from this review do not support recommending any single dietary pattern for the purpose of improving pregnancy or live birth rates in women undergoing IVF treatment.

**Supplementary Information:**

The online version contains supplementary material available at 10.1186/s12937-021-00757-7.

## Background

Approximately 15% of couples in the United States and one in four couples in developing countries are affected by infertility, defined as the inability to become pregnant after 12 months of regular unprotected intercourse [1, 2]. The World Health Organization recognizes infertility treatment and the examination of factors associated with fertility as essential to the promotion of reproductive health [1, 3].

Though in vitro fertilization (IVF) is one of the most effective treatments for infertility [4], much of the success of IVF relies on women undergoing multiple embryo transfers and oocyte retrievals. However, multiple embryo transfers and oocyte retrievals can be cost prohibitive and emotionally and physically burdensome resulting in reported treatment attrition rates of up to 35–50% [5, 6]. While some factors associated with lower success of IVF treatment, such as advanced female age, are not modifiable, there is growing interest in the impact of modifiable factors, such as diet, on treatment outcomes.

Diet currently accounts for nearly a tenth of the global burden of disease [7, 8] and epidemiological studies have linked female and male diet to reproductive outcomes. Studies on the general impact of diet on female fertility have focused largely on the examination of specific dietary nutrients and food groups, such as dairy, fats, and antioxidants, and point to several different potential pathways of effect. Animal and in vitro human cell studies indicate possible associations with mechanisms that underlie fertility including hormone levels, ovarian insufficiency, diminished ovarian reserve, and embryonic development [9–15]. Human studies link dietary factors to longer time to pregnancy and the risk of developing reproductive disorders which may impact fertility such as anovulatory infertility, endometriosis, and uterine leiomyomata [16–29]. However, despite uncovering possible links with fertility and fecundity, studies of associations between individual female dietary factors and infertility in both animals and humans are largely equivocal.

Much of what is known about the impact of individual female dietary factors specifically on IVF outcomes derives from a single observational study, the study of Environment And Reproductive Health (EARTH), described in detail elsewhere [30–32]. Within the context of IVF, female dietary patterns have been more widely studied. This reflects a trend toward viewing diet holistically in an effort to limit confounding from individual dietary items, capturing the effects resulting from the complex interactions between food groups, and providing results that are more interpretable and translatable to individuals [33–35].

To date, studies on associations between female dietary patterns and IVF outcomes have relied on observational designs. While randomized controlled trials are the gold standard for research methods, observational designs can be appropriate when the exposure is a dietary pattern; blinding may not be possible, ensuring adherence can be difficult, and participants may need to remain in a trial for long periods of time to observe the effect [36]. However, observational studies are often hindered by methodological challenges that carry the risk for bias, such as exposure misclassification, confounding control, and cohort selection. Further, observational studies are often carried out in populations and employ methods that are considerably different, necessitating careful consideration of heterogeneity across studies when comparing findings or pooling results [37, 38].

Given the importance of understanding the associations between diet, including dietary patterns, and IVF outcomes, the observational nature of existing studies, and the need to compare and conduct well-designed epidemiologic studies, we performed a systematic literature review with the following aims: to identify female dietary patterns associated with the outcomes of IVF treatment, to evaluate the body of evidence for sources of heterogeneity and methodological challenges, and to offer suggestions for minimizing heterogeneity and potential sources of bias in future studies.

## Methods

This review follows the Preferred Reporting Items for Systematic Reviews and Meta-Analysis (PRISMA) guidelines [39] (Supplemental Table 1).

### Search strategy

Articles were identified through computerized literature searches undertaken March–April 2020. We searched PubMed, EMBASE, Cochrane Central Register of Controlled Trials, and CINAHL for English language publications. In EMBASE we utilized key words and EMTREE terms: ‘infertility’ OR (‘*in vitro* fertilization’ OR ‘infertility therapy’ OR ‘IVF’) AND (‘dietary intake’ OR ‘diet’ OR ‘dietary pattern’). In PubMed we utilized MeSH terms and keywords: (‘IVF’ OR ‘*in vitro* fertilization’) OR (‘reproductive techniques, assisted’ OR ‘assisted reproductive technology’) and (‘food OR diet’). Finally, we performed a manual search of the reference lists from the final included articles.

### Selection criteria

Inclusion criteria were based on the PICO framework (Population, Intervention, Comparison, Outcome) [39]. P: women undergoing IVF or IVF with intracytoplasmic sperm injection (ICSI). Weight loss potentially improves outcomes during IVF treatment among women who are overweight (body mass index > = 25 kg/m2) or diagnosed with polycystic ovarian syndrome (PCOS) [40]; thus, we excluded studies restricted to women who are overweight or diagnosed with PCOS. I: dietary pattern with clearly delineated component food items. C: comparison group that differed in adherence to the dietary pattern. Early outcomes, such as embryo quality and yield, may not predict overall IVF treatment success [41–43]. Thus O: biochemical pregnancy (pregnancy diagnosed only by the detection of beta human chorionic gonadotrophin (βhCG) in serum or urine), clinical pregnancy (pregnancy diagnosed by ultrasonographic visualization of one or more gestational sacs or definitive clinical signs of pregnancy), or live birth (the birth of a live fetus after 22 completed weeks of gestational age) [44].

We included peer reviewed original research articles with a publication date up to March 1, 2020. Review articles, editorials, conference abstracts, opinions, and case reports were excluded.

### Assessment of study quality

The SIGN 50 Methodology Checklist 3 [45], a checklist specific to observational studies, was utilized to assess study quality. Studies were rated as “high quality” if the majority of criteria in the checklist were met and there is little risk of bias, “acceptable” if most criteria were met with some flaws and an associated risk of bias and “low quality” if either most criteria were not met or if there were significant flaws in the study design. One of the aims of this review is to explore methodological challenges in some depth. However, an initial assessment of studies was conducted to eliminate studies that did not receive a rating of “acceptable” or higher.

### Data extraction

One author extracted data from the included studies and another subsequently confirmed or disconfirmed the data. Investigators from five studies were contacted for clarification. We extracted study characteristics: first author, year of publication, location, study duration, study design (observational vs. interventional; and cross-sectional, case-control, cohort), and analytical sample size. We extracted sample characteristics: age, major exclusions, infertility diagnosis, type of ART (IVF vs IVF/ICSI), prior ART treatment, number of prior failed pregnancy attempts, and ‘duration of infertility’. We extracted data on the exposure (dietary pattern, exposure window), methods (questionnaire used for assessing exposure, timing of recruitment and exposure assessment, covariates, study end points, follow up period), and how outcomes were defined (Supplemental Table 2).

## Results

### Search results

**Fig. 1:**
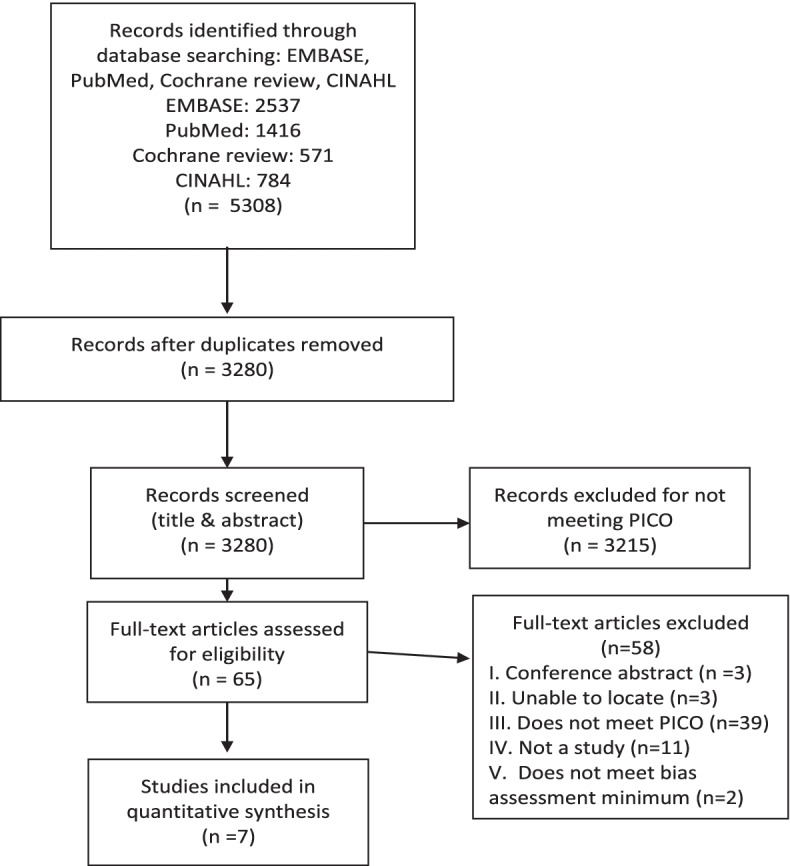
PRISMA flow diagram

The search of databases identified 5308 English language references (Fig. 1). After removal of duplicates, 3280 articles remained. We screened remaining references by reading titles and/or titles and abstracts; 3215 articles were deemed not relevant. We scanned the full texts of the remaining 65 articles and identified 56 for exclusion: 12 were reviews, opinions, or conference abstracts and 2 could not be located. 42 did not meet PICO criteria. Nine articles, representing nine independent research studies reporting on female dietary patterns and IVF outcomes, remained. No additional articles were identified after scanning reference lists.

### Assessment of study quality

Nine studies underwent the initial quality assessment and two studies were omitted; one lacked sufficient information to make an assessment [46] and one contained a measure of association for clinical pregnancy that fell outside the confidence interval (OR 0.14, 95% CI: 0.3–0.7) [47]. Study authors could not be reached to correct this error [47] (Table 1). Seven studies were rated as “acceptable” quality and were included in the review. In included studies, potential selection bias and reliability of exposure assessment were the most common inadequately addressed items (items 1.2, 1.3, 1.4, 1.5, 1.6, 1.12, Table 1).

**Table 1:** Quality assessment of studies (SIGN 50 Methodology Checklist 3)

Author (year)	1.1	1.2	1.3	1.4	1.5	1.6	1.7	1.8	1.10	1.11	1.12	1.13	1.14	2.1
Firns (2015) [46]	Yes	Can’t say	Can’t say	Can’t say	Can’t say	Can’t say	Yes	Yes	Yes	Yes	Can’t say	Yes	No	Cannot determine
Gaskins (2019) [48]	Yes	Can’t say	Yes	Does not apply	None^b^	No	Yes	Yes	Yes	Yes	No	Yes	Yes	Acceptable
Karayiannis (2018) [49]	Yes	Can’t say	No	No	None	No	Yes	Yes	Yes	Yes	No	Yes	Yes	Acceptable
Ricci (2019) [50]	Yes	Can’t say	No	No	None	No	No	Yes	Yes	Yes	No	Yes	Yes	Acceptable
Sugawa (2018) [51]	Yes	Can’t say	Yes	No	None	No	Yes	Yes	Yes	Yes	No	Yes	Yes	Acceptable
Sun (2019) [52]	Yes	Can’t say	No	No	Can’t say	No	No	Yes	No	Yes	No	Yes	Yes^a^	Acceptable*
Twigt (2012) [53]	Yes	Can’t say	No	No	None	No	Yes	Yes	No	Yes	No	Yes	Yes	Acceptable
Vujkovic (2010) [54]	Yes	Can’t say	No	Does not apply	None	No	Yes	Yes	Yes	Yes	No	Yes	Yes	Acceptable
Jahangirifar (2019) [47]														Not fully assessed ^c^

*Rated as acceptable for the primary outcome of embryo yield, low quality for biochemical and clinical pregnancy

^a^ *p*-value

^b^ All ‘in study cycles’ included, all participants had at least one ART ‘cycle’, number of women who dropped out not stated

^c^ study contained a measure of association for clinical pregnancy that fell outside the confidence interval (OR 0.14, 95% CI: 0.3–0.7)

1.1 Study addresses an appropriate and clearly focused question

1.2 Groups being studied are selected from source populations comparable in all respects other than the factor under investigation

1.3 Study indicates the number of people asked to take part who did so

1.4 Likelihood that some eligible subjects might have outcome at enrolment is assessed and considered in the analysis

1.5 Study states the percentage of individuals recruited into each arm of the study who dropped out before the study was completed

1.6 Comparison is made between full participants and those lost to follow up, by exposure status

1.7 Outcomes are clearly defined

1.8 Assessment of the outcome is made blind to exposure status

1.9 Where blinding was not possible there is recognition that knowledge of exposure status could have influenced assessment of outcome (not applicable in any study)

1.10 Method of exposure assessment is reliable

1.11 Evidence from other sources is used to demonstrate that the method of outcome assessment is valid and reliable

1.12 Exposure level or prognostic factor is assessed more than once

1.13 Main potential confounders are identified and considered in the design and analysis

1.14 Confidence intervals are provided

2. 1 Overall quality based on how well the study has done to minimise the risk of bias or confounding (high, acceptable, low)

### Dietary patterns associated with IVF outcomes

Nine dietary patterns were examined for an association with IVF outcomes in seven observational cohort studies: the MedDiet (Mediterranean diet and a ‘Mediterranean style’ dietary pattern), a Dutch ‘preconception’ diet, a ‘profertility’ diet, the ‘Fertility Diet’, the alternate Healthy Eating Index 2010 (aHEI-2010) diet, and the ‘health-conscious low processed’, ‘vegetable and seafood’, ‘Western’, and ‘rice and miso soup’ dietary patterns (Table 2).

**Table 2:** Components of dietary patterns

	Mediterranean Diet	Healthy Eating Index	Fertility Diet	profertility diet	rice and miso	vegetable and seafood	‘Western’ Diet	preconception diet	health conscious
Dietary factor		(aHEI2010)							
Seafood	+			+		+	–	+	+
Legumes	+	+							+
Fruit	+ ^k^	+		+/−^i^				+	+
Vegetables	+	+	+	+/−^i^		+		+	+
Potatoes	+^b^								
Dairy	-^e^		+/−^h^	+					
Cereal	+^c^	+		+^j^				+^j^	+^j^
Fats	+^l^	+	+				+^n^	+	
Soy				+		+			
Vitamins			+	+					
Nuts	+^d^	+							
Meat	-^f^	–	–	–			+^m^	+	–
Poultry	-^g^					+	+		
Rice/miso					+				
Alcohol	+^a^	+							

+ Indicates dietary component is used to calculate the total dietary score and contributes to a higher score

- indicates dietary component is used to calculate the total dietary score and contributes to a lower score

^a^ Sun 2019 [52] did not include alcohol

^b^ Sun 2019 [52] and Vujkovic 2010 [54] did not include potatoes

^c^ Gaskins 2019 [48], Karayiannis 2018 [49] and Sun 2019 [52] specified unrefined or whole grain; Vujkovic 2010 [54] did not include cereal

^d^ Sun 2019 [52] included nuts

^e^ Gaskins 2019 [48] and Karayiannis 2018 [49] specified full fat dairy; Vujkovic 2010 [54] did not include dairy

^f^ Vujkovic 2010 [54] did not included meat, Gaskins 2019 [48] specified red meat

^g^ Ricci 2019 [50], Sun 2019 [52], and Vujkovic 2010 [54] did not include or specify poultry

^h^ high fat dairy contributed positively, and low-fat dairy contributed negatively

^I^ low pesticide fruits and vegetables contributed positively, and high pesticide fruits and vegetables contributed negatively low pesticide fruits and vegetables contributed positively and high pesticide fruits and vegetables contributed negatively

^j^ only whole grain cereal

^k^ Vujkovic 2010 [54] did not include fruits

^l^ Vujkovic 2010 [54] specified vegetable oil, Ricci 2019 [50] high monounsaturated/saturate fatty acid ratio, Gaskins 2019 [48] and Karayiannis 2018 [49] specified olive oil

^m^ Sugawa 2018 [51] specified red meat

^n^ Sugawa 2018 [51] specified oils

#### Mediterranean diet

The MedDiet is generally comprised of high intake of whole grains, vegetables, fruits, nuts, legumes and/or pulses, and olive oil; moderate intake of nonfat or low-fat dairy products, seafood, and wine; and low consumption of poultry and red meat [55–57] (Table 2). In all five studies examining the MedDiet, participants were recruited from IVF treatment centers, had their diet assessed using a questionnaire at a point prior to embryo transfer, and followed prospectively.

Four studies examined the outcome biochemical pregnancy (Table 3). In a study of 161 couples in the Netherlands, Vujkovic (2010) reported a positive association between a positive urine pregnancy test 15 days after oocyte retrieval, and a couple’s increased adherence to a ‘Mediterranean style’ dietary pattern (adjusted OR 1.4, 95% CI: 1.0–1.9). Study participants were assigned an individual diet adherence score based on their responses to a food frequency questionnaire (FFQ). The scores from both members of the couple were then averaged and used as the exposure. No analysis was conducted to decipher associations of female or male diet alone with biochemical pregnancy. Biochemical pregnancy was the only examined pregnancy related outcome in this study and no appreciable association with biochemical pregnancy was found in any other study of the MedDiet [48, 49, 52].

**Table 3:** Associations between higher adherence to the MedDiet and outcomes

Outcome	Author (year)	Sample size	Main finding	Measure of association
**Biochemical pregnancy**	Vujkovik (2010) [54]	161 couples	+	adjusted OR (95% CI) (ref not stated): 1.4 (1.0–1.9)
	Karayiannis (2018) [49]	244 women	no appreciable association	adjusted RR (95% CI) Q1-Q3: 0.62 (0.28–1.36), 0.81 (0.39–1.65), 1.00 (ref)
	Gaskins (2019) [48]	357 women^a^	no appreciable association	adjusted proportion (95% CI) Q1-Q4: 0.49 (0.41–0.57), 0.62 (0.53–0.69), 0.64 (0.55–0.72), 0.55 (0.47–0.63)
	Sun (2019) [52]	167 women	no appreciable association	(binary) high adherence = 29.97%, low adherence = 31.75%
**Clinical pregnancy**	Karayiannis (2018) [49]	244 women	+ Age < 35 years	adjusted RR (as a continuous variable) (95% CI): Age < 35 years 1.22 (1.05–1.43) Age ≥ 35 1.00 (0.92–1.09) adjusted RR Q1-Q3 (95%CI): all women 0.35 (0.16–0.78), 0.81 (0.41–1.59), 1.00 (ref)
	Gaskins (2019) [48]	357 women^1^	no appreciable association	adjusted proportion (95% CI) Q1-Q4: 0.43 (0.35–0.50), 0.56 (0.47–0.64), 0.57 (0.48–0.66), 0.48 (0.40–0.56)
	Ricci (2019) [50]	474 women	+ Age > 35^b^	adjusted RR of not achieving pregnancy Q1-Q3 (95% CI): Age ≤ 35 1 (ref), 0.96 (0.80–1.14), 0.99 (0.81–1.20) Age > 35 1 (ref), 0.84 (0.70–1.00), 0.94 (0.81–1.20) all women 1 (ref), 0.95 (0.86–1.05), 0.98 (0.87–1.09)
	Sun (2019) [52]	167 women	no appreciable association	(binary) high adherence = 42.62%, low adherence = 50.94%
**Live birth**	Karayiannis (2018) [49]	244 women	+ Age < 35 years	adjusted RR (as a continuous variable) (95% CI): Age < 35 1.25 (1.07–1.45) Age ≥ 35 1.01 (0.93–1.11) adjusted RR Q1-Q3 (95%CI): all women 0.32 (0.14–0.71), 0.78 (0.39–1.54), 1.00 (ref)
	Gaskins (2019) [48]	357 women^1^	+ ^c^	adjusted proportion (95% CI) Q1-Q4: 0.31 (0.25–0.39), 0.47 (0.39–0.55), 0.44 (0.36–0.49), 0.41 (0.34–0.49)
	Ricci (2019) [50]	474 women	Null	adjusted RR Q1-Q3 (95% CI) Age ≤ 35 1.00 (ref), 1.00 (0.81–1.21), 1.00 (0.79–1.26) Age > 35 1.00 (ref), 0.96 (0.84–1.10), 0.97 (0.84–1.12) all women 1 (ref), 1.00 (0.90–1.11), 0.99 (0.89–1.11)

^+^ = positive association

^a^ the sample contributed 608 ART ‘cycles’ and adjustments were made for unbalanced study design (different number of cycles contributed per woman)

^b^ positive association for Q2 vs Q1 only

^c^Q2 vs Q1 only

Four studies examined associations with clinical pregnancy [48–50, 52] (Table 3). In a study conducted in a Greek population with 244 women, Karayiannis (2018) reported a positive association between high adherence to the MedDiet (as a continuous variable) and clinical pregnancy in women under the age of 35 (adjusted RR 1.2, 95% CI: 1.05–1.43). Findings were not consistent among older women (adjusted RR 1.00, 95% CI: 0.92–1.09). In a study of 474 Italian women, Ricci (2019) found an association between lower adherence to the MedDiet and risk of not achieving a clinical pregnancy however, contrary to Karayiannis (2018), the association was only present among older women aged ≥35 years. Further, among older women, the association was found only in the intermediate versus lower MedDiet adherence categories (adjusted RR 0.84, 95% CI: 0.71–1.01) and not present in the highest versus lower adherence categories (adjusted RR 0.94, 95% CI: 0.78–1.13). No other study found an appreciable association with clinical pregnancy [48, 52].

Three studies examined associations between the MedDiet and live birth [48–50] (Table 3). Gaskins and colleagues (2019) reported a positive association between the MedDiet and the probability of live birth in a sample of 357 U.S. women. There was not a dose-response association and the association was only present when comparing the lowest level of adherence to an intermediate level of adherence (probability of live birth as an adjusted proportion (95% CI) in increasing quartiles of adherence = 0.31 (0.25–0.39), 0.47 (0.39–0.55), 0.44 (0.36–0.49), 0.41 (0.34–0.49). Karayiannis (2018) found a positive association between increased adherence to the MedDiet (as a continuous variable) and live birth. Mirroring the study’s results for clinical pregnancy, the association was only among women under age 35 years (adjusted RR 1.25, 95% CI: 1.07–1.45). Ricci and colleagues (2019) reported no association with live birth in any age or dietary adherence group.

In a study conducted in China, Sun (2019) observed that clinical pregnancy rates in a high versus low adherence group was 42.62% vs. 50.94% and biochemical pregnancy rate 27.97% versus 31.75% respectively (Table 3). However, out of 590 participants, only 61 women in the high adherence group and 106 in the low adherence group had an embryo transfer by study completion. Reasons for the abbreviated follow up are not given. Results for biochemical pregnancy and clinical pregnancy were only adjusted for endometrial thickness on embryo transfer day and number of embryos transferred.

#### ‘Profertility’ diet

The ‘profertility’ diet was examined alongside the MedDiet in Gaskins 2019. The ‘profertility’ diet is based on findings from the EARTH study and comprises higher intake of supplemental folic acid, vitamin B12, vitamin D, low-pesticide fruits and vegetables, whole grains, seafood, dairy, and soy foods; and lower intake of high pesticide fruits and vegetables [32, 48] (Table 2). Higher adherence to the ‘profertility’ diet was positively associated with biochemical pregnancy, clinical pregnancy, and probability of live birth (probability of live birth as an adjusted proportion Q1 vs Q4 (95% CI) = 0.33 (0.26–0.40), 0.56 (0.47–0.64) (Gaskins, 2019) (Table 4). Findings were largely attributed to intake of micronutrients and pesticide residues on fruits and vegetables, however an indirect approximated measure of pesticide intake was used to assess exposure [48]. The sample included 357 women participating in the EARTH study and the ‘profertility’ diet has not been tested in an independent cohort [32, 48]. Gaskins (2019) followed women for multiple cycles (maximum of 6 ‘cycles’) and included all ‘in study cycles’ in the main analysis. The sample contained a relatively low number of frozen embryo transfer cycles (14%) versus fresh embryo transfer cycles (82%) when compared with recent (2016) U.S. wide treatment trends (33% frozen embryo transfer cycles versus 33% fresh embryo transfer cycles [58]).

**Table 4:** Associations between higher adherence to dietary patterns and outcomes

Dietary pattern	Outcome	Author	Sample size	Main finding	Measure of association
**‘profertility’ diet**	Biochemical pregnancy	Gaskins (2019) [48]	357 women^a^	+	adjusted proportion Q1-Q4 (95%CI): 0.46 (0.39–0.54), 0.53 (0.45–0.61), 0.65 (0.56–0.73), 0.68 (0.59–0.76)
	Clinical pregnancy	Gaskins (2019) [48]	357 women^a^	+	adjusted proportion Q1-Q4 (95%CI): 0.44 (0.33–0.48), 0.46 (0.38–0.54), 0.59 (0.50–0.68), 0.61 (0.52–0.69)
	Live birth	Gaskins (2019) [48]	357 women^a^	+	adjusted proportion Q1-Q4 (95%CI): 0.33 (0.26–0.40), 0.32 (0.25–0.40), 0.48(0.39–0.57), 0.56 (0.47–0.64)
**aHEI-2010**	Biochemical pregnancy	Gaskins (2019) [48]	357 women^a^	no appreciable association	adjusted proportion Q1-Q4 (95%CI): 0.62 (0.54–0.69), 0.59 (0.50–0.67), 0.53 (0.44–0.61), 0.54 (0.46–0.62)
	Clinical pregnancy	Gaskins (2019) [48]	357 women^a^	no appreciable association	adjusted proportion Q1-Q4 (95%CI): 0.55 (0.47–0.63), 0.51 (0.43–0.59), 0.50 (0.42–0.59), 0.45 (0.37–0.53)
	Live birth	Gaskins (2019) [48]	357 women^a^	no appreciable association	adjusted proportion Q1-Q4 (95%CI): 0.44 (0.36–0.52), 0.42 (0.34–0.50), 0.40 (0.33–0.49), 0.37 (0.29–0.45)
**‘Fertility Diet’**	Biochemical pregnancy	Gaskins (2019) [48]	357 women^a^	no appreciable association	adjusted proportion Q1-Q4 (95%CI): 0.54 (0.46–0.62), 0.58 (0.50–0.66), 0.62 (0.53–0.69), 0.54 (0.45–0.63)
	Clinical pregnancy	Gaskins (2019) [48]	357 women^a^	no appreciable association	adjusted proportion Q1-Q4 (95%CI): 0.45 (0.41–0.56), 0.53 (45–0.60), 0.52 (0.44–0.61), 0.47 (0.39–0.56)
	Live birth	Gaskins (2019) [48]	357 women^a^	no appreciable association	adjusted proportion Q1-Q4 (95%CI): 0.37 (0.30–0.45), 0.42 (0.35–0.50), 0.42 (0.34–0.50), 0.43 (0.34–0.52)
**‘health-conscious low processed’**	Biochemical pregnancy	Vujkovik (2010) [54]	161 couples	–	adjusted OR (95% CI) (ref not stated) (value provided to one decimal place in article): 0.8 (0.6–1.0)
**Dutch ‘preconception’ diet**	Clinical pregnancy	Twigt (2012) [53]	199 women	+	adjusted OR (95% CI): 1.65 (1.08–2.52)
**‘vegetable and Seafood’**	Clinical pregnancy	Sugawa (2018) [51]	140 women	no appreciable association	adjusted OR Q1-Q4 (95%CI): 1.00 (ref) 0.46 (0.14–1.53), 0.42 (0.13–1.43), 0.90 (0.30–2.69)
**‘Western’**	Clinical pregnancy	Sugawa (2018) [51]	140 women	no appreciable association	adjusted OR Q1-Q4 (95%CI): 1.00 (ref) 1.90 (0.58–6.24), 1.38 (0.41–4.61), 0.84 (0.23–3.11)
**‘rice and miso soup’**	Clinical pregnancy	Sugawa (2018) [51]	140 women	no appreciable association	adjusted OR Q1-Q4 (95%CI): 1.00 (ref), 1.78 (0.58–6.77), 1.98 (0.58–6.77), 0.72 (0.18–2.93)

+ = positive association

- = negative association.

^a^ sample contributed 608 ART ‘cycles’ and adjustments were made for unbalanced study design (different number of cycles contributed per woman)

#### A Dutch ‘preconception’ diet

In a study of 199 Dutch women undergoing IVF treatment, Twigt (2012) found a positive association between increasing adherence to a Dutch ‘preconception’ diet and ongoing pregnancy at 10 weeks (adjusted OR 1.65, 95% CI: 1.08–2.52) [53] (Table 4). The Dutch ‘preconception’ diet is comprised of: high daily intake of whole grains, vegetables, and fruit; weekly intake of at least three servings of meat or meat replacers and one serving of fish; and use of monounsaturated or polyunsaturated oils [53] (Table 2). The study occurred within the context of a preconception intervention in which women attending an outpatient OB/GYN clinic could opt into counseling to improve their lifestyle, including diet. The analytic population comprised women who opted into the intervention and subsequently underwent an IVF treatment. Findings may have different implications from other findings in this review. Participants were given a preconception dietary risk score (PDR) with the highest score corresponding to dietary intake that meets the basic requirements of a preconception diet. Thus, lower PDRs likely represent inadequate dietary intake and any increase in PDR score, a step toward adequacy. Conversely, it is not clear if lower levels of adherence to most other dietary patterns in this review correspond to an inadequate, or merely different, dietary pattern. Exposure was reassessed in 46% of participants at a voluntary follow up session, however only baseline exposure was used in the analysis.

### Dietary patterns with largely null associations with IVF outcomes

#### The aHEI-2010 diet and ‘fertility diet’

The aHEI-2010 diet and ‘Fertility Diet’ were examined alongside the MedDiet and ‘profertility’ diet in Gaskins, 2019. Higher adherence to the aHEI-2010 diet or the ‘Fertility Diet’ was not appreciably associated with biochemical pregnancy, clinical pregnancy, or live birth (aHEI-2010 diet probability of live birth as an adjusted proportion Q1 vs Q4 (95%CI) = 0.44 (0.36–0.52), 0.37 (0.29–0.45)) (‘Fertility Diet’ probability of live birth as an adjusted proportion Q1 vs Q4 (95%CI) = 0.37 (0.30–0.45), 0.43 (0.34–0.52)) [48] (Table 4). The ‘Fertility Diet’ is comprised of higher intake of monounsaturated fatty acids to trans-fat, vegetable protein, high-fat dairy, iron, and multivitamins; lower intake of animal protein and low-fat dairy; and lower glycemic load. The aHEI-2010 diet is comprised of higher intake of vegetables (excluding potatoes), fruit, whole grains, nuts and legumes, long chain omega-3 fats, polyunsaturated fat, and alcohol; and lower intake of sugar-sweetened beverages, fruit juice, red and processed meat, trans-fat, and sodium [48] (Table 2).

#### ‘Vegetable and seafood’, ‘Western’ and ‘rice and miso soup’ dietary patterns 

Like Twigt (2012), Sagawa (2018) examined the association between adherence to a ‘healthier’ dietary pattern, defined as ‘high intake of fruit and vegetables and abundant nutrients’, and IVF outcomes [51]. Sagawa identified three patterns of dietary intake in a cohort of 140 infertile Japanese women: a ‘healthier’ pattern called ‘vegetable and seafood’ with a high intake of vegetable, seafood, soy, and chicken; and two likely less healthy dietary patterns, ‘Western’ with a high intake of oil, meat, and chicken; and ‘rice and miso soup’ with a high intake of rice and miso soup (Table 2). Contrary to Twigt (2012), Sugawa (2018) found no association between higher adherence to a ‘healthier’ pattern and clinical pregnancy, confirmed by ultra sound 21 days after egg retrieval (vegetable and seafood adjusted O R per 1 category increase in adherence = 0.85 95% CI (0.67–1.39)) (‘Western’ = 0.92 95% CI (0.63–1.36))(rice and miso soup = 0.94 95% CI (0.63–1.40)) (Table 4).

#### ‘Health-conscious low processed’ dietary pattern

Vujkovik [54] examined a ‘Mediterranean style’ and ‘health-conscious low processed’ dietary pattern, within the same cohort of 161 couples. The ‘health-conscious low processed’ dietary pattern is defined as containing high intakes of fruits, vegetables, whole grains, fish, and legumes, but low intake of mayonnaise, snacks, and meat products (Table 2). Contrary to findings for the ‘Mediterranean style’ dietary pattern, a couple’s higher adherence to a ‘health-conscious low processed’ dietary pattern was associated with reduced odds of biochemical pregnancy (adjusted OR 0.8 (95% CI: 0.6–1.0) (Table 4). Vujkovik [54] attributes the difference in findings to higher intake of linoleic acid, a component found in vegetable oil, and higher levels of vitamin B6 found in the serum and follicular fluid of women with higher adherence to a ‘Mediterranean style’ dietary pattern.

### Study characteristics likely leading to increased heterogeneity

#### Study population and exclusion criteria

Studies were conducted in six countries: China, Japan, Greece, Italy, the Netherlands (*n* = 2), and U.S. (Supplement Table 2). Three studies excluded women based on underlying medical and/or reproductive conditions including; hypertension, endometriosis, or tubal factor infertility [49, 51, 54]. Two excluded older women (over 40 or 41) [49, 52], two excluded women based on treatment protocol [49, 52], and three studies contained a higher percentage of participants with male versus female factor infertility [49, 53, 54]. By exclusion criteria, one study each excluded women who did not undergo an ART treatment [48], did not undergo an embryo transfer [53], or became pregnant before treatment started [54].

#### Dietary patterns and components

Across studies, the exposure under investigation (dietary pattern) was selected using two different methods (Supplemental Table 2). In two studies, an α-posteriori approach was utilized [51, 54]. Results from participant questionnaires or FFQ were examined and the exposure was derived based on which dietary pattern best fit the data. In the remaining studies, investigators used a hypothesis driven α-priori approach. An exposure was chosen before dietary intake information was obtained and a FFQ or questionnaire appropriate for the respective pattern administered.

No two studies included the same dietary components in their definitions of the MedDiet (Table 2). All MedDiet definitions included higher intake of seafood, legumes, fruits, and vegetables. Most included low consumption of meat [48–50, 52] and low to moderate (versus no or high) intake of alcohol [48–50, 54]. Definitions inconsistently included; whole grains, type of fats and oils, dairy, nuts, poultry, and potatoes (Table 2).

#### Time period of exposure assessment

All studies reporting the exposure window period asked participants about relatively recent dietary intake with exposure windows ranging from four weeks [54] to twelve months prior to exposure assessment [48–50, 52] (Supplemental Table 2). In Sugawa (2018), participants reported their current dietary intake during the month leading up to oocyte retrieval, and in Twigt (2012) the exposure window is not stated. In two studies, women were asked whether they had changed their diet during the exposure window and were excluded if they had made a change [49, 52]. In the remaining studies, diet change during the exposure window was not reported [48, 50, 51, 53, 54].

#### Study end points and follow-up

Study length varied from one month [51] to ten years [48] (Supplemental Table 2). Participants were followed until the occurrence of at least one of the following events: biochemical pregnancy, clinical pregnancy, or live birth; completion of a maximum of six medical stimulation ‘cycles’ or treatment cessation [48], one oocyte retrieval and the transfer of resulting fresh and/or frozen embryos (only cycle with ‘best’ outcome included in analysis) [50], one oocyte retrieval and transfer of only the first fresh embryo(s) [49, 51, 53, 54], or until study end date [52]. The maximum time period between the exposure assessment and reproductive outcome was not explicitly stated across studies, however likely ranged from weeks and months [48–54] to years [48, 50], and in the case of Gaskins and colleagues (2019), potentially up to ten years.

#### Outcome definitions

Outcomes were defined somewhat inconsistently (Supplemental Table 2). Four studies reported on biochemical pregnancy defined as a rise in serum βhCG 14–21 days after oocyte retrieval [48, 49], urine test 15 days after oocyte retrieval [54], and undefined in one [52]. Six studies reported on clinical pregnancy confirmed by ultrasound at 6–10 weeks [48–51, 53], and undefined in one study [52]. Three studies reported the outcome of live birth, which was defined as birth of a neonate after 24 weeks in two studies [48, 49] and not defined in the third study [50].

### Study characteristics likely leading to methodological challenges

#### Exposure assessment

All studies utilized questionnaires to assess exposure, with most utilizing a validated self-administered semi-quantitative FFQ (number of items ranging from 6 to 131) [48, 49, 51, 54] (Supplemental Table 2). No questionnaire was validated prospectively in a population of women experiencing infertility and/or undergoing IVF treatment. In all studies, for exposure classification, participants were grouped into categories of adherence (e.g., low, intermediate, high) to the dietary pattern under investigation in relation to other participants’ adherence based on questionnaire responses. In all studies, exposure used for analyses and covariates were assessed once at baseline and not reassessed during the follow up period for changes. Studies including participants who utilized cryopreserved embryos or oocytes did not assess exposure at both the time of cryopreservation and the time of attempted use/transfer into a uterus [48, 50].

#### Timing of recruitment and exposure data collection

It is unclear if studies included baseline data on the duration of the current pregnancy attempt. A portion of participants in three studies had undergone at least one prior IVF treatment cycle during the current pregnancy attempt at the time of recruitment [48, 50, 54] while no participant had a previous IVF treatment in two studies [49, 51] (Supplemental Table 2). Four studies collected information on participants’ ‘duration of infertility’ at the time of baseline data collection [49, 52–54]. In the two studies in which a range of data was provided, participants had a mean duration of 3 years [49, 52]. Six studies collected exposure data subsequent to initial consultation for infertility; three at treatment initiation [48, 51, 52], two at the time of oocyte retrieval [49, 50] and one at the time of embryo transfer [54].

#### Covariates collected for assessment of confounding

Female age and body mass index (BMI) were the only covariates controlled for in all studies (Supplemental Table  2). All but one study controlled for energy intake and smoking [52]. ‘Duration of infertility’, previous use of ART, infertility diagnosis (male, female, unexplained), education, income, treatment protocol and use of ICSI, parity, physical activity, vitamin/supplement use, alcohol and caffeine intake, paternal covariates, and covariates related to mental health were inconsistently controlled for [48–54].

## Discussion

### Associations between dietary patterns and IVF outcomes

Nine different dietary patterns from seven observational studies were examined among participants. Higher adherence to the MedDiet, a Dutch ‘preconception’ diet, and a ‘profertility’ diet were associated with improvements in biochemical pregnancy, clinical pregnancy, or live birth in at least one study. Amongst studies of the MedDiet, findings were inconsistent and dose-response associations were only found in one study. Within the study, associations were modified by age and present only among women age < 35 and only for the outcomes of clinical pregnancy and live birth. Although examined in one relatively small population, increased adherence to a ‘profertility’ diet was associated with improvements in biochemical pregnancy, clinical pregnancy, and live birth. Likewise, higher adherence to a Dutch ‘preconception diet’ was associated with improvements in clinical pregnancy in a single small study. The aHEI-2010 diet, ‘Fertility Diet’, ‘health-conscious low processed’ dietary pattern, ‘vegetable and seafood’ dietary pattern, ‘Western’ dietary pattern, and ‘rice and miso soup’ dietary pattern were not materially associated with improved IVF outcomes. Explanations for differences in findings across and within studies on the MedDiet put forth by study authors include the escalating and overshadowing influence of age on fertility [49], lack of accounting for dietary supplements [50], and insufficient statistical power [51]. Likewise, authors of studies investigating remaining dietary patterns hypothesize that differences may be attributed to intake of substances such as pesticides [48] and linoleic acid [54]. However, causative conclusions are difficult to draw due to the high degree of heterogeneity across studies and potential bias resulting from methodological issues which may mask true associations.

### Heterogeneity across studies

Sources of heterogeneity included different study populations, dates, and length; selection of participants and dietary pattern under investigation; exposure window and assessment relative to the outcome; outcomes investigated and definitions; and control for potential confounders. However, differences in how the MedDiet was defined, geographic locations, and study end points make comparing studies especially difficult.

The MedDiet has over 34 definitions across the broader literature differing by a number of factors including constituent dietary components [59, 60]. In this review, five different MedDiet definitions were used, one determined α posteriori, and some included and/or excluded individual dietary components that have been independently associated with reproductive outcomes [31]. Moderate intake (0.5–2 glasses per day) of alcohol is a common yet controversial component of the MedDiet [61–63]. There is uncertainty around the safety of women’s alcohol consumption during conception and pregnancy [64–66], however alcohol was incorporated into almost all definitions of the MedDiet in this review. Due to the low number of studies using any one MedDiet definition, we cannot speculate on the extent to which definitional differences may have affected findings across studies. However, in future studies, it may be informative to compare analyses within a given population using different existing definitions of the MedDiet and prudent to consider excluding alcohol from future dietary pattern definitions used in studies on this topic.

Geographic location of studies may contribute to heterogeneity and affect observed associations across studies [67, 68]. Studies examining the MedDiet were conducted in five different countries, two in Mediterranean regions. As studies on the MedDiet generally contain internal comparison groups and the range of adherence differs across geographic regions, it is difficult to appreciate how the same categories of dietary intake correlate across studies. Similarly, different populations may not contain enough heterogeneity in dietary intake to fully test some hypotheses [37, 69]. In future studies, it may be useful to provide a population mean and range or clinically based cut points (when available), so that it is easier to understand how results may apply in different populations.

Lastly, study end points were heterogenous across studies. Ideally, in a study on IVF treatment, women would be followed for all pregnancy attempts until they achieved the outcome of interest or stopped treatment. Most studies on the MedDiet followed women for one fresh embryo transfer. While abbreviating the follow-up period simplifies the data collection and analysis, this strategy can oversimplify associations [70] and limit comparability. For instance, associations between exposure and the results from a single first fresh embryo transfer versus multiple embryo transfers or transfers with cryopreserved embryos, may differ. When placing findings into context, it may be helpful to limit comparisons to studies with similar end points so that women and clinicians can better interpret results.

### Methodological challenges

Three key methodological challenges of existing studies include the inaccurate assessment of exposure, enrollment of women with previous pregnancy attempts, and lack of comprehensive control for confounding.

#### Collecting accurate exposure information

Information about the potential impact of diet, healthy eating and weight loss on fertility is widely available [71–73]. Studies have reported that some women change their habits in response to unsuccessful pregnancy attempts [74–79] and that populations of women undergoing IVF treatment have a higher prevalence of disordered eating when compared with the general population [80–84].

Exposure in all studies was based on information from a single questionnaire or FFQ. However, the accuracy of FFQs and questionnaires can vary across populations [85, 86]. No study questionnaire was prospectively validated among women experiencing infertility and/or undergoing IVF treatment before use, potentially resulting in exposure misclassification. Exposure misclassification would likely attenuate associations toward the null as the outcomes was not known at the time of assessment. Even with a validated FFQ, collecting accurate information on exposure is difficult as FFQs are designed to approximate intake over a period of time. Few studies assessed if women changed their diet during the exposure window [49, 52] increasing risk for heterogeneity within categories of exposure. Future studies would benefit from FFQs prospectively validated in populations of women undergoing IVF and collecting data on any dietary changes during the exposure window.

#### Enrollment of women with previous pregnancy attempts

All studies recruited women seeking IVF treatment and it’s unclear if studies collected data on number of previous pregnancy attempts. Women seek and receive IVF treatment after a different number of pregnancy attempts and two studies included women with prior IVF attempts [87, 88]. Thus, cohorts likely contained samples with a heterogenous number of prior pregnancy attempts at baseline. If the dietary pattern under study is a cause of improved fertility, then women with higher adherence to the dietary pattern will have higher underlying fertility and will be less likely to be included in the study, resulting in a selection bias (left truncation) that could attenuate associations toward the null [89, 90]. To minimize (but not eliminate) bias from left truncation, future studies examining associations between dietary patterns and IVF outcomes could, at the very least, enroll and follow women from their initial consult at an infertility treatment center.

If infertility or a previous unsuccessful IVF cycle caused a change in diet, then reverse causation could be a potential source of bias in studies that enroll infertile couples utilizing IVF treatment. Reverse causation usually occurs in studies when participants’ knowledge of the outcome influences their exposure [91]. Although all exposure data was collected prior to the outcome, there is the potential for reverse causation if participants had related outcomes and believe their exposure may be related to these outcomes [23, 92, 93]. Women have reported using diet to enhance IVF treatment success since as early as 2001 [73, 75, 94, 95] and no data was collected in any study in this review regarding participants’ knowledge of the associations between diet and reproductive outcomes. The potential for reverse causation could be assessed in future studies by collecting data on participants’ knowledge of the potential associations between diet, fertility, and IVF outcomes, and if any change in diet was related to their knowledge.

#### Controlling for confounding

While all studies in this review collected data on potential confounders, it is difficult to anticipate and collect data on every possible confounder related to diet. Most authors acknowledge the potential for residual confounding. However, potentially important sources of residual confounding not addressed in most studies include male diet, which often mirrors female diet [96–99]; complementary and ‘add-on’ therapies, which may be used by women in conjunction with diet to enhance fertility [78, 100–105]; and weight loss [106, 107]. At a minimum, all studies should collect a broad range of data on potential confounders including demographic factors (e.g., race, ethnicity, age, and country of origin), socioeconomic position (e.g., education, occupation, income, and marital status), behavioral factors, lifestyle, anthropometrics, multivitamin use, medication use, and medical, and reproductive history. In addition, all studies should control for total energy intake to adjust for confounding, reduce measurement error, and account for differences in basal metabolic rate and body size.

### Limitations

Limitations to our systematic review should be noted when considering its findings. Across the literature, no study investigated long-term dietary patterns, therefore results only reflect recent intake. We included only English language publications in our search and excluded studies with samples restricted to women who were overweight and/or diagnosed with PCOS. A prospective registration was not undertaken and a single author conducted the literature search and screen. A meta-analysis was not conducted due to the high degree of heterogeneity across studies and the low number of studies examining any one dietary pattern and any one outcome. We did not discuss potential limitations of different statistical approaches and some findings from included studies may be spurious. Conversely, strengths of our review include the systematic approach and focus on sources of heterogeneity and bias. Likewise, our review included all study dates during the literature search and all identified dietary patterns that fit the review criteria.

## Conclusions

The literature on associations between female diet and fertility is rapidly expanding. This review adds to the current knowledge by highlighting: female dietary patterns that have been investigated for associations with IVF outcomes, ways in which studies differ, methodological challenges, and strategies that could be employed in future studies. Although some studies reported positive associations between female dietary patterns and IVF outcomes, causation cannot be assumed. Studies were potentially hindered by methodological challenges (misclassification of exposure, left truncation, and lack of comprehensive control for confounding) with an associated risk of bias. In particular, studies of the MedDiet were highly heterogenous in study population, methods, and findings, and remaining dietary patterns have each only been examined in single and relatively small populations of women. Future studies with rigorous and more uniform methodologies are needed to determine the association between female dietary patterns and IVF outcomes. At the clinical level, findings from this review do not support recommending any single dietary pattern for the purpose of improving pregnancy or live birth rates in women undergoing IVF treatment.

## Supplementary Information

**Additional file 1.** PRISMA 2009 Checklist.

**Additional file 2: Supplementary Table 2**. Characteristics of studies on female dietary patterns and IVF outcomes.

## Data Availability

Not applicable.

## Abbreviations

IVF: in vitro fertilization
ART: Assisted Reproductive Technology
ICSI: Intracytoplasmic Sperm Injection
βhCG: beta Human Chorionic Gonadotrophin
PCOS: Polycystic Ovarian Syndrome
EARTH: the study of Environmental and Reproductive Health
MedDiet: Mediterranean Diet and ‘Mediterranean style’ Dietary pattern
aHEI-2010: alternate healthy eating index 2010
FFQ: Food Frequency Questionnaire
PDR: Preconception Dietary Risk Score

## References

[1] Rutstein SO, Shah IH. Infecundity, infertility, and childlessness in developing countries. Demographic and Health Surveys (DHS) comparative reports no. 9 [Internet]. World Health Organization. World Health Organization; 2014. [cited 2022 Jan10]. Available from: https://www.who.int/reproductivehealth/publications/infertility/DHS_9/en/.

[2] Thoma ME, McLain AC, Louis JF, King RB, Trumble AC, Sundaram R (2013). The prevalence of infertility in the United States as estimated by the current duration approach and a traditional constructed approach. Fertil Steril.

[3] World Health Organization. Infertility [Internet]. World Health Organization. World Health Organization; 2019. [cited 2022 Jan 10]. Available from: https://www.who.int/health-topics/infertility#tab=tab_3.

[4] Reindollar RH, Regan MM, Neumann PJ, Levine B-S, Thornton KL, Alper MM (2010). A randomized clinical trial to evaluate optimal treatment for unexplained infertility: the fast track and standard treatment (FASTT) trial. Fertil Steril.

[5] Olivius C, Friden B, Borg G, Bergh C (2004). Why do couples discontinue in vitro fertilization treatment? a cohort study. Fertil Steril.

[6] Roest J, van Heusden AM, Zeilmaker GH, Verhoeff A (1998). Cumulative pregnancy rates and selective drop-out off patients in in-vitro fertilization treatment. Hum Reprod.

[7] Afshin A, Sur PJ, Fay KA, Cornaby L, Ferrara G, Salama JS (2019). Health effects of dietary risks in 195 countries, 1990–2017: a systematic analysis for the Global Burden of Disease Study 2017. Lancet.

[8] Lim SS, Vos T, Flaxman AD, Danaei G, Shibuya K, Adair-Rohani H (2012). A comparative risk assessment of burden of disease and injury attributable to 67 risk factors and risk factor clusters in 21 regions, 1990–2010: a systematic analysis for the Global Burden of Disease Study 2010. Lancet.

[9] Amir AA, Kelly JM, Kleemann DO, Durmic Z, Blache D, Martin GB (2018). Phyto-oestrogens affect fertilisation and embryo development in vitro in sheep. Reprod Fertil Dev.

[10] Bandyopadhyay S, Chakrabarti J, Banerjee S, Pal AK, Goswami SK, Chakravarty BN (2003). Galactose toxicity in the rat as a model for premature ovarian failure: an experimental approach readdressed. Hum Reprod..

[11] Hwang CS, Kwak HS, Lim HJ, Lee SH, Kang YS, Choe TB (2006). Isoflavone metabolites and their in vitro dual functions: They can act as an estrogenic agonist or antagonist depending on the estrogen concentration. J Steroid Biochem Mol Biol.

[12] Kuiper GGJM, Lemmen JG, Carlsson B, Corton JC, Safe SH, van der Saag PT (1998). Interaction of Estrogenic Chemicals and Phytoestrogens with Estrogen Receptor β. Endocrinology.

[13] Skaznik-Wikiel ME, Rudolph MC, Swindle DC, Polotsky AJ (2016). Elevated serum levels of biologically active omega-3 fatty acids are associated with better ovarian reserve. Fertil Steril.

[14] Swartz WJ, Mattison DR (1988). Galactose inhibition of ovulation in mice. Fertil Steril..

[15] Zhong R, Zhou D (2013). Oxidative Stress and Role of Natural Plant Derived Antioxidants in Animal Reproduction. J Integr Agric.

[16] Brasky TM, Bethea TN, Wesselink AK, Wegienka GR, Baird DD, Wise LA. Dietary Fat Intake and Risk of Uterine Leiomyomata: A Prospective Ultrasound Study. Am J Epidemiol. 2020; Available from: https://academic.oup.com/aje/advance-article/doi/10.1093/aje/kwaa097/5858261. [cited 2020 Oct 15]. 10.1093/aje/kwaa097 PMC7857646 32556077

[17] Chavarro JE, Rich-Edwards JW, Rosner B, Willett WC (2007). A prospective study of dairy foods intake and anovulatory infertility. Hum Reprod..

[18] Harris HR, Eke AC, Chavarro JE, Missmer SA (2018). Fruit and vegetable consumption and risk of endometriosis. Hum Reprod..

[19] Missmer SA, Chavarro JE, Malspeis S, Bertone-Johnson ER, Hornstein MD, Spiegelman D (2010). A prospective study of dietary fat consumption and endometriosis risk. Hum Reprod..

[20] Nodler JL, Harris HR, Chavarro JE, Frazier AL, Missmer SA (2020). Dairy consumption during adolescence and endometriosis risk. Am J Obstet Gynecol..

[21] Orta OR, Terry KL, Missmer SA, Harris HR (2020). Dairy and related nutrient intake and risk of uterine leiomyoma: a prospective cohort study. Hum Reprod Oxf Engl..

[22] Parazzini F, Chiaffarino F, Surace M, Chatenoud L, Cipriani S, Chiantera V (2004). Selected food intake and risk of endometriosis. Hum Reprod..

[23] Wesselink AK, Hatch EE, Rothman KJ, Willis SK, Orta OR, Wise LA (2020). Pesticide residue intake from fruits and vegetables and fecundability in a North American preconception cohort study. Environ Int.

[24] Willis SK, Wise LA, Wesselink AK, Rothman KJ, Mikkelsen EM, Tucker KL, et al. Glycemic load, dietary fiber, and added sugar and fecundability in 2 preconception cohorts. Am J Clin Nutr. 2020; Available from: http://academic.oup.com/ajcn/advance-article/doi/10.1093/ajcn/nqz312/5696748. [cited 2020 Jun 11]. 10.1093/ajcn/nqz312 PMC7326597 31901163

[25] Wise LA, Radin RG, Palmer JR, Kumanyika SK, Rosenberg L (2010). A Prospective Study of Dairy Intake and Risk of Uterine Leiomyomata. Am J Epidemiol.

[26] Wise LA, Radin RG, Palmer JR, Kumanyika SK, Boggs DA, Rosenberg L (2011). Intake of fruit, vegetables, and carotenoids in relation to risk of uterine leiomyomata. Am J Clin Nutr..

[27] Wise LA, Radin RG, Kumanyika SK, Ruiz-Narváez EA, Palmer JR, Rosenberg L (2014). Prospective study of dietary fat and risk of uterine leiomyomata. Am J Clin Nutr..

[28] Wise LA, Wesselink AK, Tucker KL, Saklani S, Mikkelsen EM, Cueto H (2018). Dietary Fat Intake and Fecundability in 2 Preconception Cohort Studies. Am J Epidemiol..

[29] Wise LA, Wesselink AK, Mikkelsen EM, Cueto H, Hahn KA, Rothman KJ (2017). Dairy intake and fecundability in 2 preconception cohort studies. Am J Clin Nutr..

[30] Chiu Y-H, Chavarro JE, Souter I (2018). Diet and female fertility: doctor, what should I eat?. and Sterility..

[31] Gaskins AJ, Chavarro JE (2018). Diet and fertility: a review. Am J Obstet Gynecol..

[32] Messerlian C, Williams PL, Ford JB, Chavarro JE, Mínguez-Alarcón L, Dadd R, et al. The Environment and Reproductive Health (EARTH) Study: a prospective preconception cohort. Hum Reprod Open. 2018;2018(2) Available from: https://academic.oup.com/hropen/article/2018/2/hoy001/4877108. [cited 2020 Mar 11]. 10.1093/hropen/hoy001 PMC5990043 29888739

[33] Hu FB (2002). Dietary pattern analysis: a new direction in nutritional epidemiology. Curr Opin Lipidol..

[34] Kant AK (2004). Dietary patterns and health outcomes. J Am Diet Assoc..

[35] Tapsell LC, Neale EP, Satija A, Hu FB (2016). Adv Nutr.

[36] Willett W (2013). Nutritional epidemiology.

[37] Barnard ND, Willett WC, Ding EL. The Misuse of Meta-analysis in Nutrition Research. JAMA. 2017;318(15):1435–1436. Available from: http://jamanetwork.com/journals/jama/fullarticle/2654401. [cited 2020 Jun 17] 10.1001/jama.2017.12083 28975260

[38] Eysenck HJ (1995). Meta-analysis squared—does it make sense?. Am Psychol..

[39] Moher D, Liberati A, Tetzlaff J, Altman DG, for the PRISMA Group (2009). Preferred reporting items for systematic reviews and meta-analyses: the PRISMA statement. BMJ.

[40] Broughton DE, Moley KH. Obesity and female infertility: potential mediators of obesity’s impact. Fertil Steril. 2017;107(4):840–847. Available from: https://www.fertstert.org/article/S0015-0282(17)30060-2/abstract. [cited 2019 Oct 21] 10.1016/j.fertnstert.2017.01.017 28292619

[41] Johnson NP (2006). No more surrogate end-points in randomised trials: The PCOSMIC trial protocol for women with polycystic ovary syndrome using metformin for infertility with clomiphene. Aust N Z J Obstet Gynaecol..

[42] Legro RS, Wu X, Barnhart KT, Farquhar C, Fauser BCJM, Mol B (2014). Improving the Reporting of Clinical Trials of Infertility Treatments (IMPRINT): modifying the CONSORT statement. Hum Reprod..

[43] Messerlian C, Gaskins AJ. Epidemiologic Approaches for Studying Assisted Reproductive Technologies: Design, Methods, Analysis and Interpretation. Curr Epidemiol Rep. 2017;4(2):124–132. Available from: https://www.ncbi.nlm.nih.gov/pmc/articles/PMC5636007/. [cited 2020 Feb 5] 10.1007/s40471-017-0105-0 PMC5636007 29034142

[44] Zegers-Hochschild F, Adamson GD, Dyer S, Racowsky C, de Mouzon J, Sokol R (2017). The International Glossary on Infertility and Fertility Care, 2017. Hum Reprod.

[45] Scottish Intercollegiate Guidelines Network. Checklists. SIGN. 2012. Available from: https://testing36.scot.nhs.uk. [cited 2020 Aug 21]

[46] Firns S, Cruzat VF, Keane KN, Joesbury KA, Lee AH, Newsholme P (2015). The effect of cigarette smoking, alcohol consumption and fruit and vegetable consumption on IVF outcomes: a review and presentation of original data. Reprod Biol Endocrinol RBE..

[47] Jahangirifar M, Taebi M, Nasr-Esfahani MH, Askari G (2019). Dietary Patterns and The Outcomes of Assisted Reproductive Techniques in Women with Primary Infertility: A Prospective Cohort Study. Int J Fertil Steril..

[48] Gaskins AJ, Nassan FL, Chiu Y-H, Arvizu M, Williams PL, Keller MG (2019). Dietary patterns and outcomes of assisted reproduction. Am J Obstet Gynecol..

[49] Karayiannis D, Kontogianni MD, Mendorou C, Mastrominas M, Yiannakouris N (2018). Adherence to the Mediterranean diet and IVF success rate among non-obese women attempting fertility. Hum Reprod Oxf Engl..

[50] Ricci E, Bravi F, Noli S, Somigliana E, Cipriani S, Castiglioni M (2019). Mediterranean diet and outcomes of assisted reproduction: an Italian cohort study. Am J Obstet Gynecol..

[51] Sugawa M, Okubo H, Sasaki S, Nakagawa Y, Kobayashi T, Kato K (2018). Lack of a meaningful association between dietary patterns and in vitro fertilization outcome among Japanese women. Reprod Med Biol..

[52] Sun H, Lin Y, Lin D, Zou C, Zou X, Fu L (2019). Mediterranean diet improves embryo yield in IVF: a prospective cohort study. Reprod Biol Endocrinol..

[53] Twigt JM, Bolhuis MEC, Steegers EAP, Hammiche F, van Inzen WG, Laven JSE (2012). The preconception diet is associated with the chance of ongoing pregnancy in women undergoing IVF/ICSI treatment. Hum Reprod Oxf Engl..

[54] Vujkovic M, de Vries JH, Lindemans J, Macklon NS, van der Spek PJ, Steegers EAP (2010). The preconception Mediterranean dietary pattern in couples undergoing in vitro fertilization/intracytoplasmic sperm injection treatment increases the chance of pregnancy. Fertil Steril..

[55] Panagiotakos DB, Pitsavos C, Stefanadis C (2006). Dietary patterns: A Mediterranean diet score and its relation to clinical and biological markers of cardiovascular disease risk. Nutr Metab Cardiovasc Dis.

[56] Trichopoulou A, Costacou T, Bamia C, Trichopoulos D (2003). Adherence to a Mediterranean Diet and Survival in a Greek Population. N Engl J Med..

[57] Willett WC, Sacks F, Trichopoulou A, Drescher G, Ferro-Luzzi A, Helsing E (1995). Mediterranean diet pyramid: a cultural model for healthy eating. Am J Clin Nutr..

[58] CDC. National Summary Report | 2016 ART Report | Division of Reproductive Health | CDC. 2018. Available from: https://www.cdc.gov/art/reports/2016/national-summary.html. [cited 2020 Nov 18]

[59] Bach A, Serra-Majem L, Carrasco JL, Roman B, Ngo J, Bertomeu I (2006). The use of indexes evaluating the adherence to the Mediterranean diet in epidemiological studies: a review. Public Health Nutr.

[60] Galbete C, Schwingshackl L, Schwedhelm C, Boeing H, Schulze MB (2018). Evaluating Mediterranean diet and risk of chronic disease in cohort studies: an umbrella review of meta-analyses. Eur J Epidemiol.

[61] Giacosa A, Barale R, Bavaresco L, Faliva MA, Gerbi V, Vecchia CL (2016). Mediterranean Way of Drinking and Longevity. Crit Rev Food Sci Nutr.

[62] Morales G, Martínez-González MA, Barbería-Latasa M, Bes-Rastrollo M, Gea A. Mediterranean diet, alcohol-drinking pattern and their combined effect on all-cause mortality: the Seguimiento Universidad de Navarra (SUN) cohort. Eur J Nutr [Internet]. 2020. [cited 2020 Aug 27]; Available from: 10.1007/s00394-020-02342-w. 10.1007/s00394-020-02342-w 32737614

[63] Rimm EB, Ellison RC (1995). Alcohol in the Mediterranean diet. Am J Clin Nutr.

[64] Henriksen TB, Hjollund NH, Jensen TK, Bonde JP, Andersson A-M, Kolstad H (2004). Alcohol Consumption at the Time of Conception and Spontaneous Abortion. Am J Epidemiol.

[65] Mattson SN, Schoenfeld AM, Riley EP (2001). Teratogenic Effects of Alcohol on Brain and Behavior. Alcohol Res Health.

[66] Salihu HM, Kornosky JL, Lynch O, Alio AP, August EM, Marty PJ (2011). Impact of prenatal alcohol consumption on placenta-associated syndromes. Alcohol.

[67] Kaaks R, Riboli E (1997). The role of multi-centre cohort studies in studying the relation between diet and cancer. Cancer Lett.

[68] Rosato V, Temple NJ, La Vecchia C, Castellan G, Tavani A, Guercio V (2019). Mediterranean diet and cardiovascular disease: a systematic review and meta-analysis of observational studies. Eur J Nutr.

[69] Wallström P, Sonestedt E, Hlebowicz J, Ericson U, Drake I, Persson M (2012). Dietary fiber and saturated fat intake associations with cardiovascular disease differ by sex in the Malmö Diet and Cancer Cohort: a prospective study. PloS One..

[70] Dodge LE, Farland LV, Correia KF, Missmer SA, Seidler EA, Wilkinson J, et al. Choice of statistical model in observational studies of ART. Hum Reprod. 2020; Available from: http://academic.oup.com/humrep/advance-article/doi/10.1093/humrep/deaa050/5839889. [cited 2020 Jun 27]. 10.1093/humrep/deaa050 PMC7368396 32424400

[71] Backhausen MG, Ekstrand M, Tydén T, Magnussen BK, Shawe J, Stern J (2014). Pregnancy planning and lifestyle prior to conception and during early pregnancy among Danish women. Eur J Contracept Reprod Health Care..

[72] Chavarro J (2008). The fertility diet groundbreaking research reveals natural ways to boost ovulation & improve your chances of getting pregnant.

[73] Sacha CR, Page CM, Goldman RH, Ginsburg ES, Zera CA (2018). Are women with obesity and infertility willing to attempt weight loss prior to fertility treatment?. Obes Res Clin Pract.

[74] Armour M, Sinclair J, Chalmers KJ, Smith CA. Self-management strategies amongst Australian women with endometriosis: a national online survey. BMC Complement Altern Med. 2019;19(1):–17 Available from: 10.1186/s12906-019-2431-x. [cited 2020 Jun 20]. 10.1186/s12906-019-2431-x PMC6332532 30646891

[75] Coulson C, Jenkins J. Complementary and alternative medicine utilisation in NHS and private clinic settings: a United Kingdom survey of 400 infertility patients. J Exp Clin Assist Reprod. 2005;2:–5 Available from: https://www.ncbi.nlm.nih.gov/pmc/articles/PMC1084360/. [cited 2020 Aug 20]. 10.1186/1743-1050-2-5 PMC1084360 15807886

[76] Domar AD, Conboy L, Denardo-Roney J, Rooney KL (2012). Lifestyle behaviors in women undergoing in vitro fertilization: a prospective study. Fertil Steril.

[77] Lum KJ, Sundaram R, Buck Louis GM (2011). Women’s lifestyle behaviors while trying to become pregnant: evidence supporting preconception guidance. Am J Obstet Gynecol.

[78] Stankiewicz M, Smith C, Alvino H, Norman R (2007). The use of complementary medicine and therapies by patients attending a reproductive medicine unit in South Australia: A prospective survey. Aust N Z J Obstet Gynaecol..

[79] Wise LA, Wesselink AK, Hatch EE, Weuve J, Murray EJ, Wang TR, et al. Changes in behavior with increasing pregnancy attempt time: a prospective cohort study. Epidemiology. 2020;31(5) Available from: https://journals.lww.com/10.1097/EDE.0000000000001220. [cited 2020 Jun 26]. 10.1097/EDE.0000000000001220 PMC8141253 32487855

[80] Cousins A, Freizinger M, Duffy ME, Gregas M, Wolfe BE (2015). Self-report of eating disorder symptoms among women with and without infertility. J Obstet Gynecol Neonatal Nurs JOGNN NAACOG..

[81] Freizinger M, Franko DL, Dacey M, Okun B, Domar AD (2010). The prevalence of eating disorders in infertile women. Fertil Steril.

[82] Greenwood EA, Pasch LA, Cedars MI, Huddleston HG (2020). Obesity and depression are risk factors for future eating disorder-related attitudes and behaviors in women with polycystic ovary syndrome. Fertil Steril.

[83] Stewart DE, Robinson E, Goldbloom DS, Wright C (1990). Infertility and eating disorders. Am J Obstet Gynecol..

[84] Sylvester C, Menke M, Lopa S, Gopalan P (2020). Disordered eating and distress in women seeking in vitro fertilization. J Psychosom Res.

[85] Cade J, Thompson R, Burley V, Warm D (2002). Development, validation and utilisation of food-frequency questionnaires – a review. Public Health Nutr.

[86] Thompson FE, Byers T (1994). Dietary Assessment Resource Manual. J Nutr..

[87] Jain T (2006). Socioeconomic and racial disparities among infertility patients seeking care. Fertil Steril.

[88] Missmer SA, Seifer DB, Jain T (2011). Cultural factors contributing to health care disparities among patients with infertility in Midwestern United States. Fertil Steril.

[89] Eijkemans MJC, Leridon H, Keiding N, Slama R. A Systematic Comparison of Designs to Study Human Fecundity: Epidemiology. 2019;30(1):120–9 Available from: http://journals.lww.com/00001648-201901000-00016. [cited 2020 Jun 28]. 10.1097/EDE.0000000000000916 30198936

[90] Schisterman EF, Cole SR, Ye A, Platt RW (2013). Accuracy Loss Due to Selection Bias in Cohort Studies with Left Truncation. Paediatr Perinat Epidemiol.

[91] Rothman KJ, Greenland S, Lash TL (2008). Modern Epidemiology. 3rd ed.

[92] Cnattingius S, Akre O, Lambe M, Ockene J, Granath F (2006). Will an adverse pregnancy outcome influence the risk of continued smoking in the next pregnancy?. Am J Obstet Gynecol.

[93] Tran DT, Roberts CL, Jorm LR, Seeho S, Havard A (2014). Change in smoking status during two consecutive pregnancies: a population-based cohort study. BJOG Int J Obstet Gynaecol.

[94] Porter M, Bhattacharya S (2008). Helping themselves to get pregnant: a qualitative longitudinal study on the information-seeking behaviour of infertile couples. Hum Reprod.

[95] Rossi BV, Bressler LH, Correia KF, Lipskind S, Hornstein MD, Missmer SA (2016). Lifestyle and in vitro fertilization: what do patients believe?. Fertil Res Pract.

[96] Aly JM, Polotsky AJ (2017). Paternal Diet and Obesity: Effects on Reproduction. Semin Reprod Med..

[97] Cheng PJ, Pastuszak AW, Hotaling JM (2019). Is it time to start folate supplementation in men?. The effect of paternal folate status on embryonic growth. and Sterility..

[98] Marchetti F (2011). How do paternal life style and environmental exposures impact on sperm and early embryos?. Birth Defects Res Part - Clin Mol Teratol..

[99] Oostingh EC, de Vos I, Ham AC, Brouwer-Brolsma EM, Willemsen SP, Eggink AJ (2019). No independent associations between preconception paternal dietary patterns and embryonic growth; the Predict Study. Clin Nutr..

[100] Kissin DM, Kawwass JF, Monsour M, Boulet SL, Session DR, Jamieson DJ (2014). Assisted hatching: trends and pregnancy outcomes, United States, 2000–2010. Fertil Steril.

[101] Lensen S, Shreeve N, Barnhart KT, Gibreel A, Ng EHY, Moffett A (2019). In vitro fertilization add-ons for the endometrium: it doesn’t add-up. Fertil Steril.

[102] Rayner J-A, Willis K, Burgess R (2011). Women’s use of complementary and alternative medicine for fertility enhancement: a review of the literature. J Altern Complement Med N Y N..

[103] Smith JF, Eisenberg ML, Millstein SG, Nachtigall RD, Shindel AW, Wing H (2010). The use of complementary and alternative fertility treatment in couples seeking fertility care: data from a prospective cohort in the United States. Fertil Steril.

[104] Spencer EA, Mahtani KR, Goldacre B, Heneghan C. Claims for fertility interventions: a systematic assessment of statements on UK fertility centre websites. BMJ Open. 2016;6(11) Available from: https://bmjopen.bmj.com/content/6/11/e013940. [cited 2020 Jan 16]. 10.1136/bmjopen-2016-013940 PMC5168515 27890866

[105] de Lacey SL, Sanderman E, Smith CA (2018). Acupuncture in reproductive medicine: the motivations of infertile women to participate in a randomised controlled trial. J Psychosom Obstet Gynaecol..

[106] Chavarro JE, Ehrlich S, Colaci DS, Wright DL, Toth TL, Petrozza JC (2012). Body mass index and short-term weight change in relation to treatment outcomes in women undergoing assisted reproduction. Fertil Steril.

[107] Moran L, Tsagareli V, Norman R, Noakes M (2011). Diet and IVF pilot study: Short-term weight loss improves pregnancy rates in overweight/obese women undertaking IVF. Aust N Z J Obstet Gynaecol..

